# Denial of Pregnancy in a Patient With a History of Pseudocyesis

**DOI:** 10.7759/cureus.14773

**Published:** 2021-04-30

**Authors:** Matthew R Narlesky, Fahim Rasul, Suporn Braaten, Andrew Powell, Robert G Wooten

**Affiliations:** 1 Psychiatry, Unity Health, Searcy, USA; 2 Research, Unity Health, Searcy, USA

**Keywords:** mental health in pregnancy, antipsychotics during pregnancy, schizophrenia, pseudocyesis, denial of pregnancy, pregnancy denial, pregnancy delusion, substance abuse, ob/gyn, delusions

## Abstract

Denial of pregnancy is a condition in which a pregnant patient does not believe she is pregnant. This case describes a 23-year-old Caucasian female, with a past psychiatric history of pseudocyesis, stimulant use disorder, and schizophrenia, who was admitted to the inpatient psychiatric unit for the treatment of psychosis, suicidal thoughts, and homicidal ideation. During her hospitalization, an intrauterine pregnancy was confirmed with three serum quantitative human chorionic gonadotropin (hCG) levels and a transabdominal ultrasound. Despite definitive evidence of pregnancy, the patient reported it was impossible she was pregnant and stated she had not had intercourse for more than a year. The patient was treated with IM haloperidol decanoate and PO haloperidol. Care was coordinated with the obstetrics team to ensure the patient and her fetus received adequate prenatal care. After acute stabilization, the patient was discharged with close follow-up. This case presentation describes one of the few documented cases of pregnancy denial in a patient with a history of pseudocyesis. Additionally, this case highlights the ethical issues associated with the treatment of pregnancy denial patients. Additional studies are necessary to fill in the gaps in the literature on this unique condition.

## Introduction

Denial of pregnancy is a condition in which a pregnant patient does not believe she is pregnant. Some literature suggests that denial of pregnancy stems from a response to fears and doubts related to pregnancy; denial, a primitive defense mechanism, is thought to ameliorate the patient’s emotional conflict and anxiety through an unconscious rejection of reality [1,2]. The pregnancy denial can become so severe that the patient develops unawareness of her pregnancy, resulting in a failure to develop an attachment to the fetus and prepare for delivery [1-3]. Denial of pregnancy is believed to exist along a continuum, spanning from uncertainty about the pregnancy to genuine unawareness of the pregnancy even in the face of irrefutable evidence [2,4]. Often the presence and severity of denial vary at different times during the pregnancy [4].

The literature generally classifies denial of pregnancy as psychotic or non-psychotic [2]. In psychotic denial of pregnancy, the patient tends to be chronically mentally ill and is often psychotic for the duration of the pregnancy [1]. Patients with non-psychotic denial of pregnancy typically do not have a history of psychotic illness and often exhibit intact reality testing outside of their pregnancy denial [1]. Some authors have further categorized non-psychotic pregnancy denial as pervasive, affective, or persistent [1]. Pervasive denial is characterized by the patient experiencing unawareness of the pregnancy while affective denial is characterized by an intellectual awareness of the pregnancy but a lack of physical or emotional preparation for the birth [1]. Persistent denial involves the patient learning of her pregnancy in the third trimester yet not seeking antenatal care [1]. In the interest of completeness, it is worth noting that pseudocyesis, a condition characterized by the belief of being pregnant despite medical evidence to the contrary, exists on the opposite side of the spectrum of pregnancy-related delusions [5].

Denial of pregnancy is a relatively uncommon condition. A study conducted at obstetric hospitals and midwives' practices in Berlin found the incidence of pregnancy denial to be 1 in 475, while a study at an American academic medical center reported the incidence to be 1 in 516 [6,7]. Although research has not identified a well-defined set of characteristics in pregnancy denial patients, current research indicates the majority of patients who develop denial of pregnancy are multiparous and do not have a history of psychiatric illness [7-9].

There currently is not a consensus regarding the treatment of patients with denial of pregnancy [1]. Because of denial of pregnancy's associated risks, including increased rates of neonaticide, child neglect, and postpartum emotional disturbance, evaluation by a psychiatrist is prudent [1,10]. Psychotherapy is one of the primary treatments for denial of pregnancy [11,12]. In terms of pharmacological treatment, antipsychotics are understood to reduce the severity of the pregnancy denial, but data is limited on treatment specifics [12]. Denial of pregnancy should be managed by an interdisciplinary team as treatment often includes pharmacotherapy, psychotherapy, evaluation of parenting skills and support network, and obstetric care [1,11].

The ethics of treating a patient with denial of pregnancy are also an important consideration. The question of whether the patient has the capacity to make her own decisions must be addressed. If the patient is determined to lack capacity, then the treatment team must consider how to act in the best interest of the mother and fetus, which may entail evaluating legal options to aid in treating the patient [1].

## Case presentation

This report describes the case of a 23-year-old Caucasian female, with a past psychiatric history of pseudocyesis, stimulant use disorder, and schizophrenia, who was admitted to the inpatient psychiatric unit for treatment of auditory hallucinations that commanded her to harm herself and others. Work-up in the emergency department prior to admission was significant for a positive serum human chorionic gonadotropin (hCG) and a urine drug screen positive for amphetamine. Although the patient reported she was prescribed amphetamine salts, a query of the prescription monitoring program did not corroborate the patient’s claims. During the patient’s psychiatric evaluation, she was uncooperative and minimized her symptoms, stating she did not need psychiatric treatment. She appeared guarded and paranoid, often responding to questions by stating “that is personal.”

As per collateral (the patient’s mother), the patient had been living at a local homeless shelter prior to admission. The patient had a nine-month-old child whom she had been prohibited from contacting by the Department of Human Services. The patient’s mother reported that the patient had a history of sexual abuse perpetrated by her brother; the patient’s mother also reported the patient was not married. She voiced concerns about the patient's history of medication noncompliance and reported that the patient had assaulted her father a few weeks prior to her admission.

On the first day of her present admission, the treatment team carefully weighed the risks and benefits of treating the patient's psychosis given her positive serum hCG result. The team reached a consensus that the patient's psychosis was a significant danger to both the patient and her fetus; the team subsequently reviewed the patient's medication history and evaluated treatment options. The patient was started on haloperidol 2 mg p.o. q.a.m. and haloperidol 5 mg p.o. q.h.s. Two additional serum quantitative hCG levels revealed increasing levels consistent with pregnancy. After five days of treatment, the patient continued to be verbally aggressive and delusional, voicing a bizarre conspiracy about not being discharged so the treatment team could inappropriately observe her in the shower. Because of her persistent psychotic symptoms, her haloperidol dose was increased to 5 mg p.o. q.a.m. and 5 mg p.o. q.h.s. The treatment team repeatedly attempted to counsel the patient about her pregnancy and the need for prenatal care, but she continued to be resistant to any additional evaluation of her pregnancy.

On the sixth day of hospitalization, the treatment team reached out to an obstetrician/gynecologist (OB/GYN) who the patient claimed had cared for her during her last pregnancy; however, the physician did not have any record of ever having treated the patient. The team consulted the hospital OB/GYN department; the staff OB/GYN recommended a transabdominal ultrasound to confirm and, if indicated, date an intrauterine pregnancy. Despite medication adjustment the previous day, the patient continued to be markedly psychotic, easily agitated, and verbally aggressive.

On hospital day 7, the patient became irate when discussing her pregnancy. She was resolute that she had not had intercourse in more than a year and that pregnancy was impossible. She reported her hCG levels were elevated because she had a special blood type that made it appear she was pregnant. Of note, the patient had claimed her blood type had led to an erroneous pregnancy test result during a prior admission for pseudocyesis, although at that time she claimed it had led to a false negative.

At this point, the team was presented with an ethical dilemma regarding whether to evaluate legal options for initiating treatment. Although the patient had demonstrated some degree of compliance on the unit, the patient had a history of chronic noncompliance in all aspects of outpatient treatment and continued to deny she was pregnant. For the safety of the patient and her fetus, the team pursued a 45-day court order for treatment. The treatment team felt this was in the best interest of both the patient and the fetus, particularly given the severity of the patient’s psychosis when she is noncompliant with her psychiatric medications.

**Figure 1:**
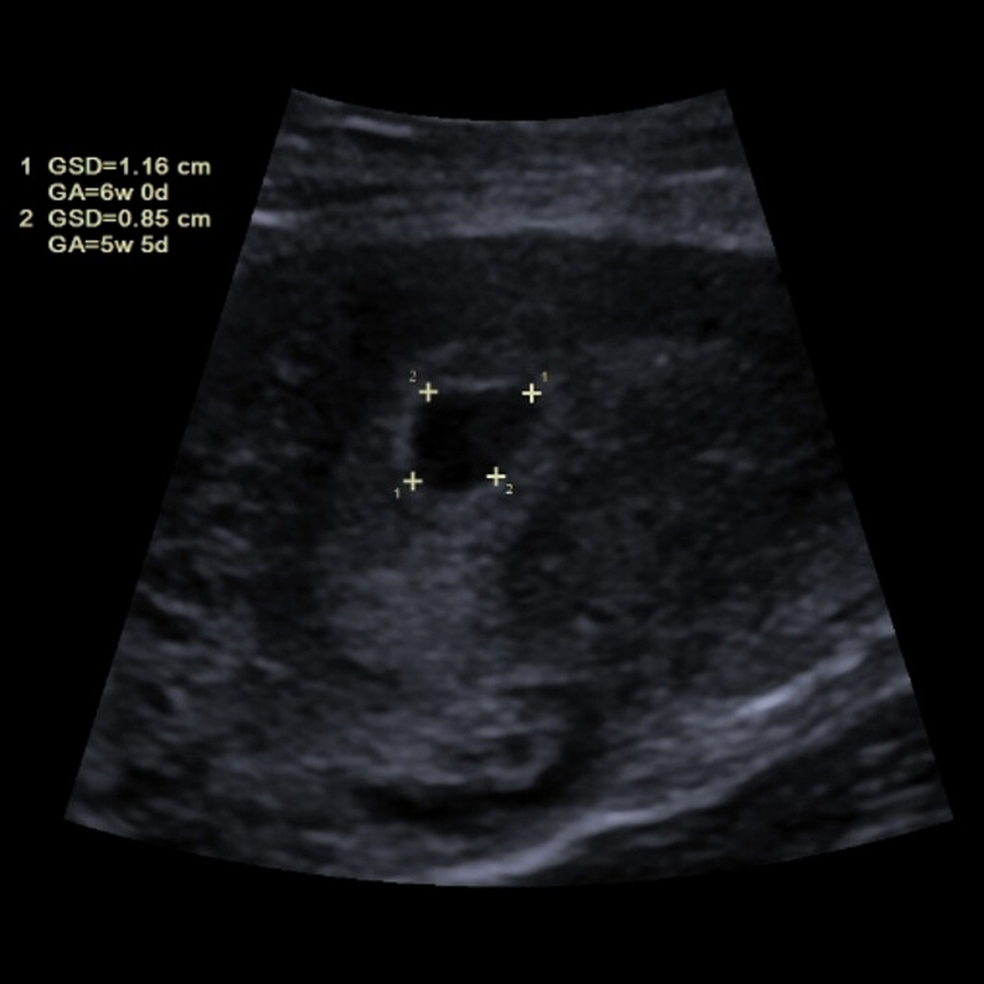
Transabdominal ultrasound showing the gestational sac.

On hospital day 8, a transabdominal ultrasound (Figure 1) confirmed an intrauterine gestation with an average age of five weeks and six days. Despite the unequivocal results, the patient continued to deny having had sexual intercourse in more than a year and maintained it was impossible she was pregnant.

On hospital day 9, the patient appeared in court, and a 45-day court order for treatment was granted. The treatment team began working on a suitable discharge plan. The patient perseverated on returning to the homeless shelter she had lived at prior to admission. The treatment team felt discharging to a facility with more resources was in the best interest of the patient; however, due to limitations resulting from the COVID-19 pandemic, it was evident the patient’s only option was to return to the homeless shelter. The treatment team reached out to the director of the homeless shelter who was agreeable to accommodating the patient’s needs and ensuring she received the necessary treatment. Because of her history of chronic noncompliance, the patient was administered haloperidol decanoate 100 mg IM the following morning.

On hospital day 16, the patient was discharged. She was discharged on haloperidol decanoate 100 mg IM monthly and haloperidol 5 mg p.o. b.i.d. At the time of discharge, she was cordial and cooperative. Her hallucinations, suicidal ideation, and homicidal ideation had all resolved. She was no longer responding to internal stimuli and was optimistic about her future. She still was intermittently in denial of her pregnancy, although she was agreeable to obstetric treatment. The patient was discharged with appointments for therapy, medication management, and obstetric follow-up.

## Discussion

This case addresses several gaps in the current literature on denial of pregnancy. One unique aspect of our case is the patient was diagnosed with denial of pregnancy early in her pregnancy while much of the literature describes patients who were diagnosed retrospectively, often at the time of delivery [13]. Another reason this case contributes to the literature on denial of pregnancy is because of the nature of the patient’s denial. The patient, in this case, exhibited unwavering denial, which is in contrast with the vacillation between denial and awareness frequently seen in psychotic denial of pregnancy [12]. Additionally, to our knowledge, this is one of a few documented cases of a patient who has exhibited both pseudocyesis and denial of pregnancy.

Our case also highlights the need for further understanding of pharmacologic treatments in denial of pregnancy. Antipsychotics are generally understood to decrease the severity of the denial; however, data are limited on the specifics of using antipsychotics to treat the condition [12]. The literature often references psychotropics without disclosing details of the treatment [1,2]. We opted to include the medication, dose, and frequency in our case to provide readers with a specific clinical example of pregnancy denial treatment. When considering antipsychotic treatment in a pregnant patient, providers must weigh the potential increase in teratogenic risk against the risk of untreated psychosis [14]. The literature on antipsychotic treatment during pregnancy is not robust enough to draw definitive conclusions; however, the available data suggest that first-generation antipsychotics are the treatment of choice in drug-naïve patients while patients currently on an efficacious antipsychotic therapy should be continued on that therapy [14]. Similarly, there is a lack of information about the use of specific psychotherapy modalities in treating denial of pregnancy, although some research suggests supportive psychotherapy may be beneficial [12].

Additionally, this patient presentation demonstrates the importance of early intervention in denial of pregnancy. Although research has not identified well-defined risk factors to inform screening for denial of pregnancy, some authors have theorized a benefit from having a low threshold for pregnancy testing women of child-bearing age who present with symptoms suggestive of pregnancy [1]. One study found that 38% of pregnancy denial patients had been evaluated by a physician during pregnancy and not received a diagnosis of pregnancy [8]. In our case, the patient was screened for pregnancy while in the emergency department, and the OB/GYN department was consulted early during the patient's hospitalization. Similarly, this case emphasizes the importance of an interdisciplinary approach to treatment. Coordination of care between the psychiatry team, social work, and the obstetrician was necessary to ensure the patient received timely management of both her mental illness and pregnancy.

This case also underscores the need for careful consideration of the ethical issues associated with the treatment of patients with denial of pregnancy. In this case, pursuing a court order for treatment was in the best interest of the patient and her fetus. When making these decisions, clinicians must evaluate the patient’s capacity and weigh patient autonomy against the potential of the patient not receiving treatment. When evaluating the long-term care of the patient, clinicians should consider the patient's parenting capabilities. Unfortunately, the outcomes of children born to pregnancy denial patients are not well-characterized. A review of available data on children born to pregnancy denial patients found that approximately half of the cases resulted in the death of the neonate while half of the remaining cases involved placement of the infant in adoption or foster care [2].

Finally, our case report highlights the need for further research on the diagnosis of denial of pregnancy. It is generally understood that the diagnosis involves an unawareness of pregnancy in a pregnant patient; however, specific criteria evaluating other domains of clinical relevance, such as the chronicity of symptoms, have not been established [2,13]. When the pregnancy is objectively confirmed and the patient exhibits denial, the primary differential diagnosis is pregnancy concealment, which occurs when the patient is aware of her pregnancy but attempts to hide it from others [8]. There has also been discussed in the literature as to whether pregnancy denial should be included in the *Diagnostic and Statistical Manual of Mental Disorders* [2,15-17]. Some authors have called for denial of pregnancy to be classified as an adjustment disorder because the condition involves a maladaptive reaction to an external stressor [2]. Some have taken this a step further and recommended including the diagnosis in a proposed subtype of adjustment disorder called maladaptive denial of physical illness [15-17]. Alternatively, Beier et al. opine that denial of pregnancy could be classified as a sexual and gender identity disorder because the symptoms are related to reproductive experience and behavior [8]. As the subtypes of pregnancy denial are associated with different outcomes, further research should explore the clinical value of further categorizing denial of pregnancy [1].

## Conclusions

Denial of pregnancy is an uncommon condition that complicates both the patient’s psychiatric and obstetric treatment. An interdisciplinary approach to treatment is necessary to ensure the patient receives comprehensive care. Psychotherapy and antipsychotics are mainstays of treatment. Because of the nature of the delusion, ethical considerations will often arise during the course of treatment. In severe cases, the provider may need to pursue a court order for treatment. Current literature presents a basic framework for the disorder, but there is much to be learned about the diagnosis, treatment, and prognosis of the condition.
